# Significance of fatty acid metabolism regulators in the diagnosis and subtype classification in non-alcoholic fatty liver disease

**DOI:** 10.3389/fcell.2026.1871166

**Published:** 2026-06-19

**Authors:** Ziyi Cao, Runzhi Yu, Ying Cheng, Wenyan Fei, Yuanwen Chen

**Affiliations:** 1 Department of Gastroenterology, Huadong Hospital Affiliated to Fudan University, Shanghai, China; 2 Shanghai Key Laboratory of Clinical Geriatric Medicine, Shanghai Institute of Geriatrics and Gerontology, Shanghai, China

**Keywords:** consensus clustering, diagnostic biomarker, fatty acid metabolism, non-alcoholic fatty liver disease, subtype

## Abstract

**Introduction:**

Non - alcoholic fatty liver disease (NAFLD) is currently the most common steatosis of the liver among Chinese. Nevertheless, a large proportion of individuals go undiagnosed and receive no treatment because of the absence of dependable and efficient diagnostic methods. Given the vital part that fatty acid metabolism (FAM) dysregulation plays in the development of NAFLD, this research aimed to assess the possible worth of FAM regulators as diagnostic biomarkers for NAFLD.

**Methods:**

GSE89632 (24 normal liver tissue samples and 39 NAFLD liver tissue samples) was utilized as the training cohort. FAM regulators were extracted from the Molecular Signatures Database. The four most crucial FAM regulators, demonstrating the highest significance (FABP4, CPOX, TLR2, and OXT), as identified by the RF model, were used to develop the diagnostic nomogram. To validate the nomogram, analyses of the calibration curve, decision curve (DCA), and clinical impact curve were carried out. FAM subtypes were categorized according to crucial FAM regulators, and genes with differential expression (DEGs) associated with FAM were pinpointed between the subtypes. Further division of gene subtypes was based on FAM-related DEGs.

**Results:**

Results: The RF model achieved a higher area under the curve (AUC) than the SVM model in diagnostic ROC analysis. FAM subtype B exhibited higher macrophage infiltration, and gene subtype B consistently exhibited higher infiltration levels of immune cells. FAM scores were also remarkably elevated in FAM subtype B and gene subtype B compared with corresponding FAM subtype A and gene subtype A.

**Discussion:**

The aforementioned genes were then validated by external datasets (GSE48452, GSE66676, and GSE135251). Finally, the expression of key genes was verified in the HepG2 cells and mice. In summary, these findings support the feasibility of using FAM regulators to predict NAFLD occurrence.

## 1 Introduction

The key pathophysiological hallmark of non-alcoholic fatty liver disease (NAFLD) is a high accumulation of lipids in the liver. This condition is triggered by a variety of factors, with alcohol and other well - defined liver - damaging agents being excluded (Siddiquee et al., 2015). In modern society, the improvement in living quality and the shift in dietary patterns have led to an increase in obesity. Obesity is recognized as a definite risk factor for NAFLD, with the associated morbidity in obese patients reported at approximately 40%∼90% (Fan et al., 2017). As a progressive liver ailment, the natural course of NAFLD encompasses non-alcoholic simple fatty liver, non-alcoholic steatohepatitis, progressive liver fibrosis, and eventual cirrhosis (Powell et al., 2021). This progression unfolds over an extended period, and a substantial number of patients find themselves at the irreversible advanced stage upon initial diagnosis, posing a substantial disease burden on the individual and society (Younossi, 2019). The absence of any specific symptoms and reliable markers makes the early diagnosis of NAFLD challenging. Therefore, the exploration of reliable diagnostic biomarkers of NAFLD is of paramount importance as it may significantly improve diagnosis and intervention strategies, resulting in a more favorable clinical outcome.

The pathogenesis of NAFLD is complex, with the “double strike theory” the most commonly accepted theoretical framework (Cobbina and Akhlaghi, 2017; Yang Y. et al., 2021). According to the theory, there are two phases of disease development. The first phase is characterized by increased fat content in cells as a result of increased intake of fats into cells and accelerated production of fatty acids. Mainly, the primary factor in the development of the disease is the disturbed fatty acid metabolism (FAM) regulation, which results from the malfunctioning of fatty acid metabolism regulators, including genetic factors involved in fatty acid transport (such as the FABP family), production, oxidation, and lipid droplet formation. Failure to maintain proper regulation leads to fat accumulation and thus to metabolic disorders (Ipsen et al., 2018). When hepatocytes fail to store the mass fatty acids in the form of triglyceride or face an overload in cellular oxidation, the high incidental reactive oxygen species may induce endoplasmic reticulum stress, oxidative stress, inflammation, and apoptosis (Xi et al., 2025; Lou et al., 2025; Chuang et al., 2025), collectively representing the second strike (Farzanegi et al., 2019; Nasiri-Ansari et al., 2021). One of the regulatory enzymes involved in the metabolism of fatty acids and also known to synthesize heme is coproporphyrinogen oxidase (CPOX). The role of coproporphyrinogen oxidase is oxidative decarboxylation in heme synthesis. Heme is an important compound required for the formation of cytochrome and hemoprotein for respiration and oxidation. Oxidative stress results from CPOX malfunction, resulting in heme synthesis disorder. Therefore, the disorder in fatty acid metabolism (FAM) is pivotal in the onset and advancement of NAFLD (Alves-Bezerra and Cohen, 2018). Deciphering the diagnostic value of FAM regulators may provide new perspectives, contributing to advancements in the understanding and identification of NAFLD, ultimately leading to more effective diagnostic methods.

The use of machine learning has proven very effective in many different applications (Vu-Thi-Minh et al., 2026; Dinh-Quoc et al., 2025; Ta et al., 2025b). Particularly in biomedical sciences, there has been tremendous success using machine learning to detect patterns and biomarkers that have not been previously noticed using conventional statistics. This is especially important in the study of NAFLD, which is a disease that lacks efficient non-invasive means for diagnosis. Machine learning algorithms like random forest and support vector machines are capable of detecting patterns in high-dimensional data sets (Bui-Minh et al., 2025; Le, 2025; Ta et al., 2025a) and thus have proven highly effective in many biomedical applications.

Herein, the diagnostic value of FAM regulators was assessed in NAFLD by establishing a utilizable diagnostic model. The initial screening of the cells for a quartet of genes that regulate inflammation was successful and resulted in a resolution of a trio of genes that may offer potential diagnostic tools for hepatic steatosis. Furthermore, this study was successful in classifying NAFLD into two different subtypes based on key FAM regulators, which were characterized by distinct FAM activity and infiltration patterns of immune cells. This may help to better understand the heterogeneity of NAFLD.

## 2 Materials and methods

### 2.1 Data processing

The GSE89632 dataset (GPL570 platform), containing 24 healthy liver control tissues and 39 NAFLD liver tissue samples are obtained from the Gene Expression Omnibus (GEO) database. Raw data were normalized using the robust multi-array average (RMA) method, and no significant batch effects were detected by principal component analysis. Furthermore, a comprehensive set of 68 pathways or biological processes related to fatty acid metabolism was sourced from curated gene sets (C2 collection), ontology gene sets (C5 collection), and hallmark gene sets (H collection), in the Molecular Signatures Database (MSigDB). Additionally, a TXT format file, consolidating the data of these biological mechanisms, pathways, and associated genes, was established. The file encompassed 700 genes. Please refer to Supplementary Material 1 for further details.

### 2.2 Identification of differentially expressed fatty acid metabolism regulators

Differentially expressed genes (DEGs) were identified from the 700 FAM regulators via the DESeq2 R package (v 1.30.0). Genes with |Log2FoldChange| > 1 and adjusted P < 0.05 were regarded as DEGs. Subsequently, all the FAM regulators were subjected to Gene Set Enrichment Analysis (GSEA) functional enrichment analysis using the fgsea R (v 1.10.1) package.

### 2.3 Development of gene co-expression network

The transcriptome dataset from GSE89632 was employed to build a weighted co - expression network using the WGCNA R package. For further analysis, genes that had the top 25% in terms of median absolute deviation were retained. The integrity of the data was confirmed using the goodSampleGenes function, and the most suitable soft threshold (β) was chosen through the pickSoftThreshold method. The matrix data then underwent a transformation into an adjacency matrix. As per the computed topological overlap, the different gene modules were clustered. The module eigengene was then calculated with the subsequent merging of the modules in the clustering tree that exhibited similarity, leading to the depiction of a hierarchical clustering dendrogram. To evaluate the importance of genes and clinical data, gene and module significance were calculated. Additionally, correlations between several modules and the normal samples and NAFLD were examined. Furthermore, calculating the module membership for each gene aided in assessing the corresponding gene significance.

### 2.4 Establishment of the random forest model and support vector machine model

To identify FAM regulators for subsequent analysis, the grey module from WGCNA, which exhibited the strongest correlation with NAFLD and the highest gene significance, was intersected with differentially expressed FAM regulators. Although the grey module in WGCNA typically contains unassigned genes, it was biologically meaningful in this dataset and was therefore retained for further analysis. To find possible diagnostic indicators for NAFLD, two models were created: a support vector machine (SVM) model and a random forest (RF) model (Le and Xuan-Thi-Thu, 2024; Ngoc et al., 2024). The randomForest R package was used to create the RF model with 100 trees (ntree = 100) and three randomly chosen variables each split (mtry = 3) (Slimene et al., 2026; Hsu et al., 2025; Li X. et al., 2024; Ran et al., 2024). Ten-fold cross-validation was carried out inside the training set for parameter tuning and to reduce overfitting after the dataset was randomly divided into a training set (70%) and a validation set (30%) (Le et al., 2020). The SVM model was constructed using the e1071 R package with a radial basis kernel, and its hyperparameters (cost and gamma) were optimized via grid search. Key FAM regulators were subsequently selected using ten-fold cross-validation. The e1071 R package (v 1.6.7) was used to construct the SVM diagnostic model. Additionally, the models were evaluated using boxplots of residuals, the diagnostic receiver operating characteristic (ROC) curve, and the reverse cumulative distribution of residuals.

### 2.5 Construction of the diagnostic nomogram

Using the “rms” package in R, we created a nomogram predictive of the four hub genes, which performed the best in our RF model. We performed decision curve analysis (DCA) and plotted clinical impact.

### 2.6 Classification of FAM subtypes in NAFLD based on pivotal FAM regulators

In the ConsensusClusterPlus R package, we identified subtypes of the FAM in NAFLD based on FAM regulators. We confirmed the clustering by principal component analysis (PCA). The expression profiles of pivotal FAM regulators in FAM subtypes were also determined.

### 2.7 Identification of FAM-related differentially expressed genes between FAM subtypes

DEGs between FAM subtypes were determined by the DESeq2 R package and were named FAM-related DEGs. Genes with |Log2FoldChange| > 0.585 and adjusted P < 0.05 were categorized as DEGs. A lower fold-change threshold was applied because this analysis compared two FAM subtypes within the NAFLD population. Employing the clusterProfiler R package (version 3.6.0), we conducted functional enrichment analyses on these FAM - associated differentially expressed genes (DEGs). The functional annotations were performed using the Gene Ontology (GO) and the Kyoto Encyclopedia of Genes and Genomes (KEGG) databases.

### 2.8 Classification of gene subtypes in NAFLD based on FAM-related differentially expressed genes

Further division of patients with NAFLD into various gene subtypes was carried out utilizing the FAM-related DEGs via the ConsensusClusterPlus R package. The expression patterns of FAM-related DEGs and pivotal FAM regulators in gene subtypes were further determined.

### 2.9 Calculation of fatty acid metabolism score

The FAM score for each individual in the training cohort was calculated using the PCA algorithm. Specifically, the FAM score was determined as = PC1i, wherein PC1 represents the principal component 1, and i represents the level of expression of FAM-related DEGs.

### 2.10 Evaluation of immune enfiltration

The infiltration landscape of 23 immune cell populations was estimated via the single-sample gene set enrichment analysis (ssGSEA) and their correlations to FAM regulators across the training group. Furthermore, the variation in the infiltration level of these aforementioned cells within the FAM subtypes or gene subtypes was assessed.

### 2.11 Cell culture and treatment

HepG2, the human hepatocellular carcinoma cell line, procured from the Cell Bank of the Chinese Academy of Sciences (Shanghai, China), was cultivated in DMEM medium (Gibco; Thermo Fisher Scientific, Inc., Waltham, MA, USA). The medium was supplemented with 1% penicillin/streptomycin (Gibco; Thermo Fisher Scientific, Inc.) and 10% fetal bovine serum (FBS) (ScienCell Research Laboratories, Inc., San Diego, CA, USA). The cells were cultured at 37 °C in an incubator with 5% CO2. To trigger steatosis, HepG2 cells, at a confluence of 80%, were subjected to a 24-h treatment with 0.25 mM palmitic acid. The controls were designated as the cells maintained in 2% BSA and DMEM for 24 h.

### 2.12 Oil red O staining and cellular triglyceride assays

Oil Red O (ORO) staining was utilized for the visualization and quantification of the lipid droplets. Following the provided protocol, formalin was utilized for cell fixation for a duration of 10 min, with a subsequent 20-min exposure to the newly prepared ORO stain. This was followed by a wash with 60% isopropanol and restaining via Mayer hematoxylin for a duration of 2 min. A light microscope (NIKON, Japan) was utilized to observe and image the resulting red-stained lipid droplets at ×100 and ×200 magnification. To analyze the intracellular lipid accumulation, the levels of TG (triglycerides) and TC (total cholesterol) were examined in HepG2 cells using a commercially recommended assay kit for TG and TC content.

### 2.13 Animal experiments

To establish an NAFLD model, C57BL/6J mice (male, 8 weeks old, JSJ Laboratory) were divided into two groups (n = 12) and fed with a chow diet and a high-fat diet, respectively. The mice were kept under SPF conditions, with a 12-h light/dark cycle, an ambient temperature of 23 °C ± 3 °C, and a relative humidity of 35% ± 5%. They were housed on corn cob bedding and had free access to the aforementioned two diets. The feeding period lasted for 12 weeks, and blood samples and liver tissues were collected for testing at the end of the experiment. Animal experiments were approved by the Institutional Animal Care and Use Committee of Fudan University (Approval No. 2022JS-Huadong hospital-067).

### 2.14 Liver histopathological examination

To carry out Oil Red O staining, frozen sections with a thickness of 6 μm were prepared. These sections were then treated with a freshly diluted solution of this stain for a duration of 20 min. Afterward, the sections were washed with 60% isopropanol with subsequent restaining via Mayer hematoxylin for a period of 2 min. The samples were observed and imaged under a light microscope to assess their histological characteristics.

To utilize the hematoxylin and eosin (H&E) stain, the liver tissue underwent fixation in 4% paraformaldehyde for a duration of 48 h. Following fixation, the tissue was transferred to 70% alcohol to facilitate subsequent histological investigations. Paraffin was used for embedding the tissue, and sections with a thickness of 3 μm were cut before removing the paraffin. Subsequently, these sections were stained with hematoxylin-eosin reagents.

### 2.15 Polymerase chain reaction

Palmitic acid was utilized to stimulate HepG2 cells, mimicking the microenvironment of steatosis. Hub genes mRNA levels were assessed by Quantitative real-time PCR (qRT-PCR). The mRNA relative expression of FABP4, CPOX, TLR2, and OXT was detected after 24 h of incubation. The extraction of the total RNA of HepG2 cells was executed per the provided protocol by utilizing the Cell Lysis Buffer (EZBioscience, USA). Using the PrimeScript™ RT Master Mix from Takara Bio, located in Shiga, Japan, cDNA was synthesized on a thermal cycler (Applied Biosystems PE4500, originating from the USA). After synthesis, the generated cDNA products were stored at 4 °C to be used for subsequent expression analysis. A quantitative real - time polymerase chain reaction (qRT - PCR) was carried out within a 20 - mL reaction setup. For this operation, the One Step SYBR® PrimeScript™ RT - PCR Kit II from TaKaRa, a Chinese company, was utilized. Table 1 showcases the primer sequences that were used in the qRT - PCR.

**TABLE 1:** The sequence fragments of RNAs.

Gene	Forward primer sequence	Reverse primer sequence
FABP4	CCT​TAG​ATG​GGG​GTG​TCC​TG	TCG​TGG​AAG​TGA​CGC​CTT​TC
CPOX	CGT​CCA​AGG​AGG​AGG​TGT​TT	TGT​GCC​CCG​ATC​ATA​CAG​CA
TLR2	CCA​AGT​GAA​GGC​AGG​AAG​ACA	GGA​AAC​TCG​AGG​CAG​ACC​AA
OXT	ACT​TGA​TGG​CTC​CGA​ACA​CC	TTT​CAC​CAT​TTC​TGG​GGT​GG

### 2.16 Western blotting analysis

For the extraction of total proteins, a RIPA lysis buffer (Beyotime, P0013B) was prepared on ice. This buffer was supplemented with 1 mM PMSF and 1 mM phosphatase inhibitors (Abcam, ab201112). After the protein extraction, the samples were mixed with SDS loading buffer and subjected to denaturation at 100 °C for 15 min. After denaturation, the protein lysates were separated on SDS-PAGE and electroblotted on nitrocellulose membranes. Immunoblots were conducted using an overnight probe of the primary antibody at 4 °C, followed by a one-hour probe of the respective secondary antibodies at room temperature. To detect the immunoreactive signals, the ECL system was employed. These signals were visualized using a BioRad imager.

### 2.17 Immunohistochemistry

Immunohistochemical analyses required deparaffinization of paraffin sections for antigen repair, followed by blocking with 10% BSA and incubating with corresponding antibodies, and finally using DAB staining.

### 2.18 External dataset validation

The GSE48452 dataset (GPL11532 platform) contains 14 healthy liver control tissues and 32 NAFLD liver samples, the GSE66676 dataset (GPL6244 platform) contains 34 healthy liver control tissues and 26 NAFLD liver tissue samples, and the GSE135251 dataset (GPL18573 platform) contains 10 healthy liver control tissues and 206 NAFLD liver tissue samples. All data were obtained from the GEO database. Normalization method that maintained consistency between three external validation datasets and the training set GSE89632. Verify the differential expression of four key genes (FABP4, CPOX, TLR2, OXT) using these three datasets.

### 2.19 Statistical analyses

Statistical inquiries were conducted utilizing the R software, specifically version 4.0.3. To conduct comparisons among different groups, the Kruskal - Wallis test was utilized. For parametric analyses, two - tailed tests were utilized. Pearson correlation analysis was applied to examine correlations. To account for multiple testing, P-values were adjusted using the Benjamini–Hochberg (BH) false discovery rate method. A correlation coefficient with an absolute value (∣r∣) greater than 0.1 and BH-adjusted P < 0.05 was considered statistically significant and biologically meaningful. Regarding significance levels, “*” represents P ≤ 0.05, “**” indicates P ≤ 0.01, and “***” stands for P ≤ 0.001.

## 3 Results

### 3.1 Overview of fatty acid metabolism regulators in NAFLD

**FIGURE 1:**
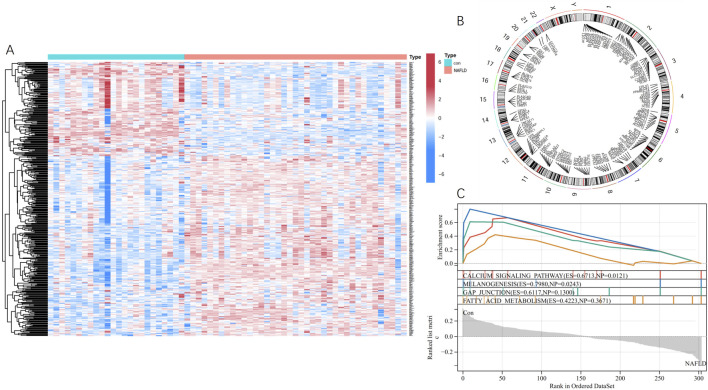
Landscape of FAM regulators in NAFLD. **(A)** Expression pattern of the differentially expressed FAM regulators between NAFLD samples and normal samples. **(B)** Chromosomal locations of the differentially expressed FAM regulators. **(C)** GSEA functional enrichment analysis of all the FAM regulators.

In the training cohort, 402 differentially expressed FAM regulators were identified between NAFLD samples and the normal (Figure 1A). The chromosomal location of these differentially expressed FAM regulators was displayed (Figure 1B). GSEA functional enrichment analysis primarily depicted that these FAM regulators also participate in the calcium signaling pathway, melanogenesis, and gap junction (Figure 1C).

### 3.2 Gene co-expression network and gene modules from WGCNA

**FIGURE 2:**
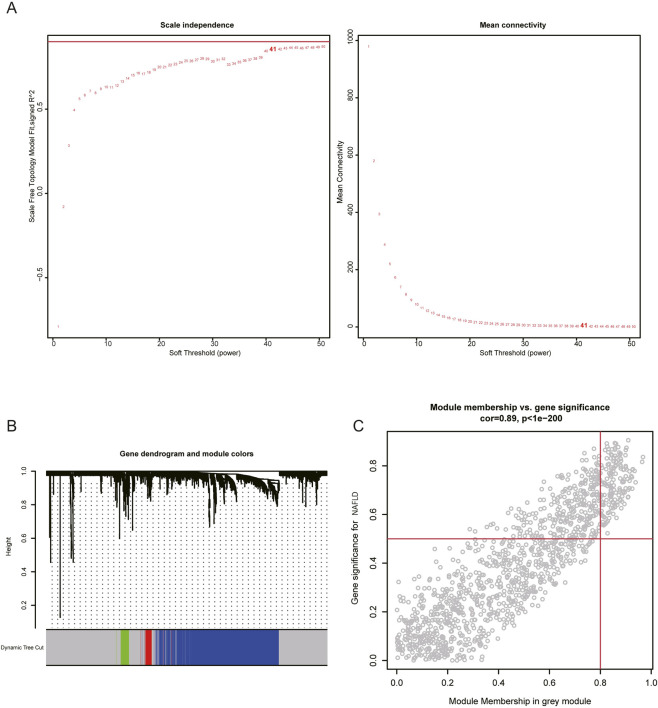
Evaluation of soft-thresholding power in WGCNA and modules. **(A)** Examination of the scale-free fit index and the mean connectivity across different soft-thresholding powers (β). The red line denotes a correlation coefficient of 0.9, with the associated soft-thresholding power identified as 41. **(B)** The cluster dendrogram of the genes exhibiting median absolute deviation in the top 25%. Each branch in the figure signifies an individual gene, while each color below signifies a co-expression module. **(C)** Scatter plot illustrating the association of the module membership with gene significance within the grey module.

20,790 genes were used to construct the gene co-expression network. The pickSoftThreshold function was used to assess soft-thresholding powers ranging from 1 to 50. To ensure a biologically meaningful scale-free network topology, a power of 41 was chosen. Since it was the lowest value that produced a scale-free R2 > 0.8 and a slope approximating −1.5 (Figure 2A). The co-expression matrix was subsequently established using the one-step method, and dynamic hybrid cutting was applied to identify four gene modules (Figure 2B). The correlations between these four modules and NAFLD or normal samples were visualized. The gray module, which is composed of 1,204 genes, demonstrated the most robust correlation with NAFLD (r = −0.83, P = 4E−17) and had the highest gene significance. Moreover, a substantial correlation was detected between gene importance and module affiliation within the gray module (r = 0.89, P < 1E−200) (Figure 2C). Consequently, the gray module was selected for further investigation.

### 3.3 The random forest model and support vector machine model

**FIGURE 3:**
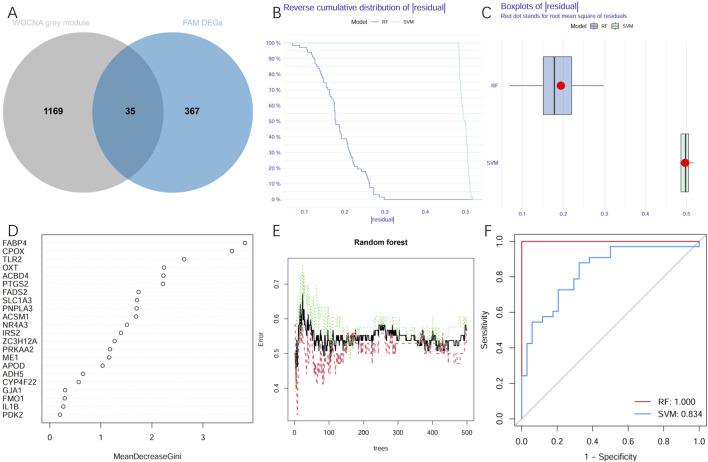
Construction of the RF model and SVM model. **(A)** Venn diagram between differentially expressed FAM regulators and the grey module from WGCNA. **(B)** Reverse cumulative distribution of the residual of the RF model and the SVM model. **(C)** Boxplots of the residuals of the RF model and the SVM model. **(D)** The top 22 pivotal FAM regulators from the RF model. **(E)** Random forest tree validation of the RF model. **(F)** Diagnostic ROC curves of the RF model and SVM model.

The differentially expressed FAM regulators and grey module from WGCNA underwent intersection, resulting in the identification of 35 genes (Figure 3A). An assessment using the reverse cumulative distribution of residuals and residual box-plots demonstrated that the Random Forest (RF) model had minuscule residuals (Figures 3B,C). Most of the samples in the RF model showed comparatively small residuals, which signified its superiority. Therefore, the RF model was selected as the ultimate model for forecasting the onset of NAFLD. The 22 pivotal FAM regulators were displayed according to their significance (Figure 3D). The RF model was further validated (Figure 3E). Moreover, diagnostic ROC curves revealed that the RF model has a more favorable area under the curve (AUC) compared with the SVM model (Figure 3F).

### 3.4 The diagnostic nomogram for NAFLD

**FIGURE 4:**
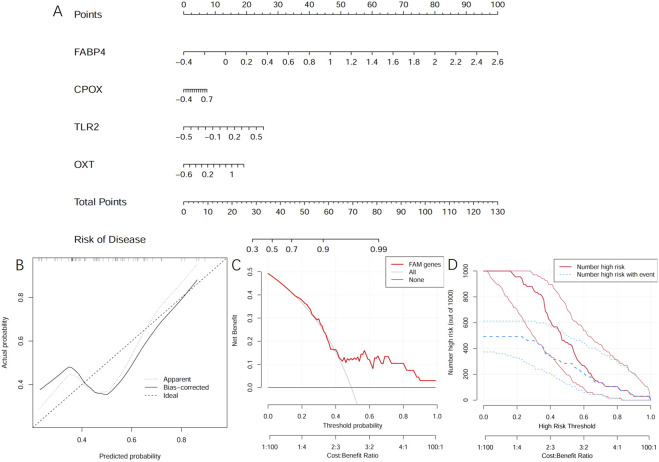
Diagnostic nomogram for NAFLD based on four FAM regulators. **(A)** Nomogram integrating FABP4, CPOX, TLR2, and OXT to predict the probability of NAFLD. Each regulator contributes to the total point score, which corresponds to the predicted risk. **(B)** Calibration curve demonstrating agreement between predicted and observed probabilities. **(C)** Decision curve analysis showing net benefit across threshold probabilities. **(D)** Clinical impact curve illustrating the number of patients classified as high risk by the nomogram compared with the actual number of cases.

The top four pivotal FAM regulators (FABP4, CPOX, TLR2, and OXT) (Xu et al., 2025; Li E. et al., 2024) from the RF model were applied to construct the diagnostic nomogram (Figure 4A). FABP4 was determined to be the strongest predictor according to its full contribution to the cumulative points. The fair predictive accuracy of the nomogram was identified by calibration curves (Figure 4B). The DCA revealed the evident benefit of the diagnostic nomogram at a certain threshold probability (Figure 4C). The clinical impact curves depicted the favorable prognostic capability of the nomogram (Figure 4D).

### 3.5 Two FAM subtypes were classified in NAFLD based on pivotal FAM regulators

**FIGURE 5:**
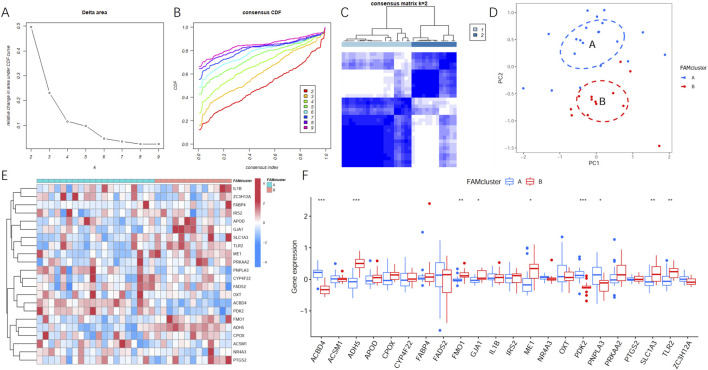
Consensus clustering of patients with NAFLD based on 22 pivotal FAM regulators. **(A)** Association between the relative changes in the area under the cumulative distribution function (CDF) curve and the number of subtypes (k value). **(B)** Consensus CDF of the different numbers of clusters. **(C)** Consensus clustering when k = 2. **(D)** PCA of the two FAM subtypes. **(E)** Expression pattern of the 22 pivotal FAM regulators in FAM subtype A and FAM subtype B. **(F)** Differential expression of the 22 pivotal FAM regulators between FAM subtype A and FAM subtype B.

**FIGURE 6:**
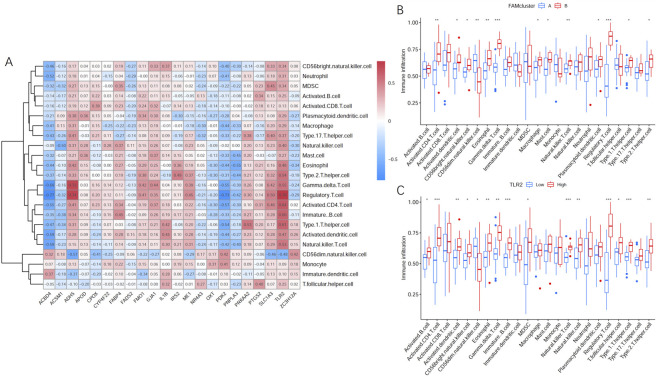
Single-sample gene set enrichment analysis of immune cells. **(A)** Correlation between the 22 pivotal FAM regulators and 23 immune cells. **(B)** Differential infiltration levels of 23 immune cells between FAM subtype A and FAM subtype B. **(C)** Differential infiltration levels of 23 immune cells between high and low TLR2 expression subgroups.

Consensus clustering revealed that it achieves the optimal classification when two subtypes are divided (k = 2) (Figures 5A–C). PCA verified the two distinct FAM subtypes in NAFLD (Figure 5D). The expression pattern of the 22 pivotal FAM regulators in two FAM subtypes was displayed (Figure 5E). The expression levels of ADH5, FMO1, GJA1, ME1, SLC1A3, and TLR2 were significantly higher in FAM subtype B, while ACBD4, PDK2, and PNPLA3 exhibited a remarkably higher expression level in FAM subtype A (Figure 5F). Correlation analysis indicated that TLR2 had a significant positive association with numerous immune cell types (Figure 6A). Moreover, the infiltration pattern of immune cells varied between FAM subtype A and FAM subtype B (Figure 6B). In FAM subtype B, the infiltration levels of activated CD4 T cells, activated dendritic cells, CD56bright natural killer cells, eosinophils, γδ T cells, macrophages, regulatory T cells, natural killer T cells, mast cells, plasmacytoid dendritic cells, type 1 Th cells, and type 2 Th cells were elevated. In contrast, only the infiltration level of CD56dim natural killer cells was higher in FAM subtype A. Furthermore, it was found that patients with high TLR2 expression commonly exhibit more abundant immune infiltration (Figure 6C).

### 3.6 Two gene subtypes of NAFLD were identified according to FAM-related differentially expressed genes

**FIGURE 7:**
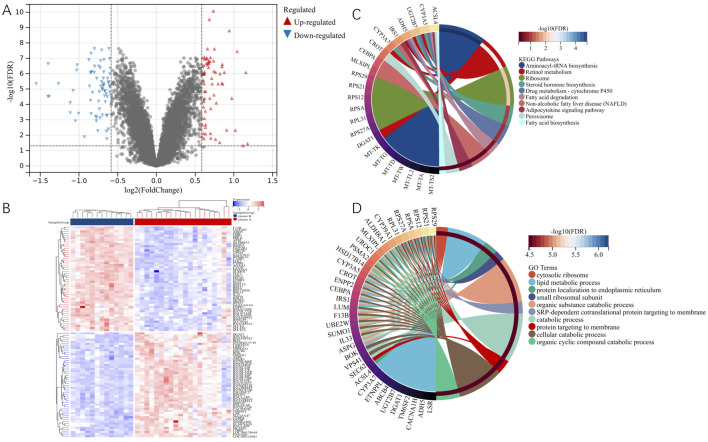
Determination of 142 FAM-related DEGs and functional enrichment analysis. **(A)** Volcano plot of the 142 FAM-related DEGs. **(B)** Expression pattern of the 142 FAM-related DEGs between FAM subtype A and FAM subtype B. **(C)** KEGG functional enrichment analysis of the 142 FAM-related DEGs. **(D)** GO functional enrichment analysis of the 142 FAM-related DEGs.

142 FAM-related DEGs between FAM subtype A and FAM subtype B were determined and displayed (Figures 7A,B). GO and KEGG functional enrichment analysis exhibited that these FAM-related DEGs are significantly associated with metabolic activities, including cellular RNA biosynthesis, steroid hormone biosynthesis, lipid-related catabolic activities, and particularly, the NAFLD (Figures 7C,D). Subsequently, patients with NAFLD were again divided into different gene subtypes based on the 142 FAM-related DEGs by consensus clustering, and two gene subtypes achieved the optimal classification. The expression pattern of the 142 DEGs linked to FAM in the two gene subtypes was displayed in Supplementary Figure S1. As for the 22 pivotal FAM regulators, the expression levels of ADH5, FMO1, GJA1, ME1, and TLR2 were significantly higher in gene subtype B, while the expression levels of ACBD4, PDK2, and PNPLA3 were higher in gene subtype A. Additionally, the infiltration level of most of the immune cells was elevated in gene subtype B.

### 3.7 Patients with NAFLD can be discriminated by pivotal FAM regulatory pattern

**FIGURE 8:**
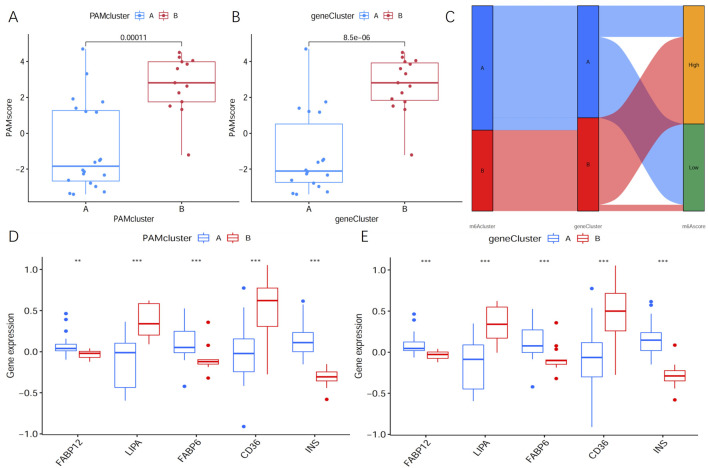
Role of pivotal FAM regulatory pattern in distinguishing NAFLD. **(A)** Difference of FAM score between FAM subtype A and FAM subtype B. **(B)** Variation in FAM score between gene subtypes A and B. **(C)** The distribution among FAM subtypes, gene subtypes, and high/low FAM score by Sankey diagram. **(D)** Differential expression levels of FABPs (FABP12, LIPA, FABP6, CD36, and INS) between FAM subtype A and FAM subtype B. **(E)** Differential expression levels of FABPs (FABP12, LIPA, FABP6, CD36, and INS) between gene subtypes A and B.

To further characterize the pivotal FAM regulatory pattern, the FAM score of each NAFLD sample was calculated. As a result, patients in FAM subtype B or gene subtype B carried FAM scores that were notably elevated in comparison with the patients in FAM subtype A or gene subtype A (Figures 8A,B). The distribution among FAM subtypes, gene subtypes, and high/low FAM scores was also visualized (Figure 8C). To further analyze the association between FAM regulatory pattern and NAFLD, the expression levels of fatty acid-binding protein (FABPs) (FABP12, LIPA, FABP6, CD36, and INS) were comparatively assessed between different FAM subtypes and gene subtypes. The resulting data indicated that the expression levels of FABP12, FABP6, and INS are remarkably higher in FAM subtype A or gene subtype A, while the expression levels of LIPA and CD36 are significantly higher in FAM subtype B or gene subtype B (Figures 8D,E).

### 3.8 The validation of hub biomarkers

**FIGURE 9:**
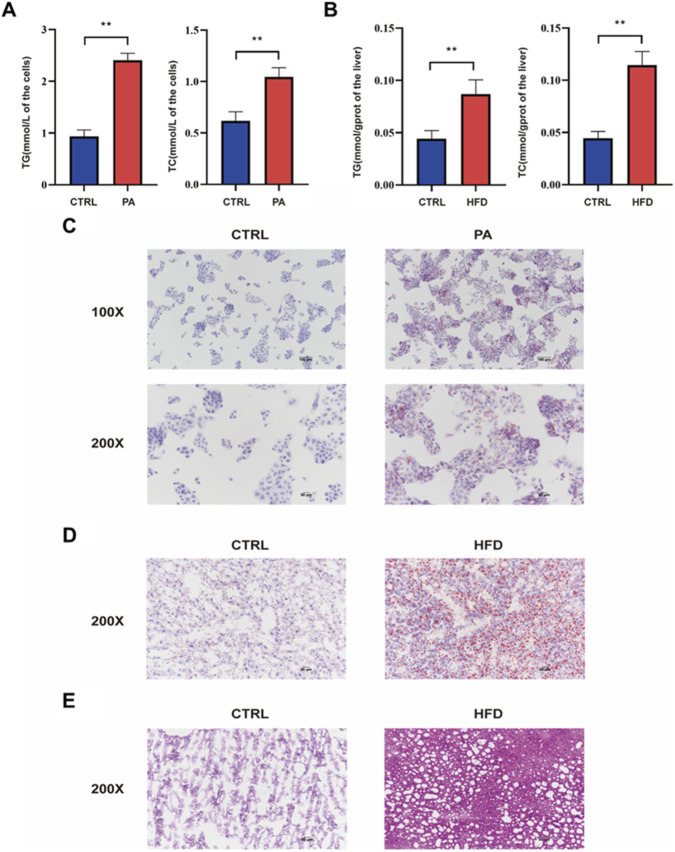
Establishment of cell and animal high-fat models. **(A)** TG and TC levels of HepG2 cells. **(B)** TG and TC levels in the liver of control and HFD mice. **(C)** Oil red O staining of HepG2 cells. **(D,E)** Oil red O staining (×200) and HE staining (×200) of the liver.

**FIGURE 10:**
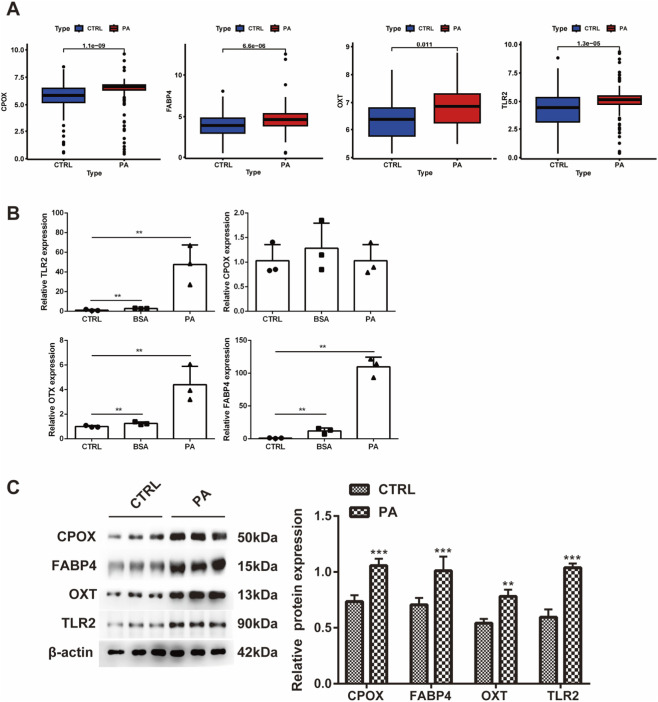
Verification of 4 FAM regulators. **(A)** Validation of hub genes performance in external datasets. **(B)** The relative expressions of hub genes were validated by qRT-PCR. **(C)** Western blot analysis and quantification of hub genes. Data are represented as mean ± SD, ***p < 0.001, **p < 0.01.

**FIGURE 11:**
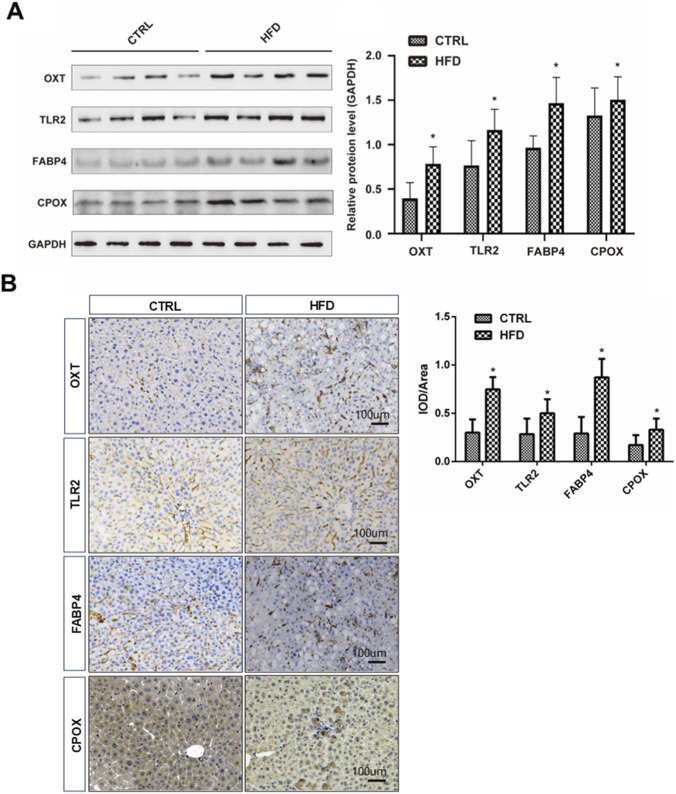
Biochemical indices and protein levels. **(A)** Key protein levels and quantification in the liver of control and HFD mice. **(B)** Immunohistochemical results of key proteins. Data are represented as mean ± SD, **p < 0.01, *p < 0.05.

To verify the accuracy of the four genes mentioned above, we established *in vitro* and *in vivo* fatty liver models to examine their expression and used three liver tissue sequencing datasets (GSE48452, GSE66676, and GSE135251) as external validation sets. The establishment effect of HepG2 steatosis and the fatty liver mouse model was shown in Figure 9. The results showed that both PA intervention and HFD diet successfully increased TG and TC levels in cell lines and mouse liver (Figures 9A,B) and significantly induced lipid droplet formation (Figures 9C–E). We included a total of 356 samples from three datasets, consisting of 85 healthy liver tissues and 271 fatty liver tissues (detailed in Supplementary Material 3). After merging the expression matrices and sample group information, batch effect correction was performed, followed by differential expression analysis of the four key genes. The expression levels of CPOX (P = 1.1 × 10^−9^), FABP4 (P = 6.6 × 10^−6^), OXT (P = 0.011), and TLR2 (P = 1.3 × 10^−5^) all showed statistically significant differences between groups (Figure 10A). The RNA levels of the four genes were detected in HepG2 cells after modeling, and the results showed that the mRNA levels of FABP4, OXT, and TLR2 were upregulated in the PA group, while the expression of CPOX was not significantly different (Figure 10B). However, the protein levels of all four genes were significantly upregulated in the PA group (Figure 10C). To further validate the expression of four central genes, Western blotting was performed in mouse liver tissue, and the results showed that the protein levels of the four genes were upregulated in the HFD group (Figure 11A). The immunohistochemical staining results of the mouse liver were consistent with the WB results (Figure 11B).

## 4 Discussion

In China, NAFLD is common among patients with fatty liver disease, yet its prevalence is often underestimated, resulting in poor awareness of the associated disease burden (Zhou et al., 2020). Many patients remain undiagnosed and untreated due to a lack of effective diagnostic tools (Zhou et al., 2020). Given the critical role of fatty acid metabolism disorder in NAFLD pathogenesis (Alves-Bezerra and Cohen, 2018), few studies have investigated the diagnostic potential of fatty acid metabolism (FAM) regulators in this context. Accordingly, this study sought to determine whether FAM-associated regulatory genes could serve as viable predictive tools for NAFLD diagnosis.

In total, 402 differentially expressed FAM regulators were identified between NAFLD samples and the normal samples. Utilizing WGCNA, the extent of candidate genes was further narrowed. Four pivotal FAM regulators (FABP4, CPOX, TLR2, and OXT) with the highest significance were finally screened by the RF model to construct a diagnostic nomogram for predicting NAFLD occurrence. The fair distinguishing capability of the diagnostic nomogram was verified via calibration curves and clinical impact curves. Negative outcomes in patients with NAFLD or hepatocellular carcinoma (HCC) have been associated with the fatty acid binding protein 4 (FABP4) (Coilly et al., 2019; Thompson et al., 2018). Also, the induction of liver cancer stem cells from hepatocytes during the conversion of non-alcoholic steatohepatitis (NASH) to liver cancer was also identified by Yang H. et al. (2021). Toll-like receptor genes were commonly activated in chronic liver disease, including NAFLD (Roh and Seki, 2013). Toll-like receptor 2 (TLR2) is an important regulator in propelling hepatic injury, inflammation, lipid accumulation, fibrosis, and steatosis (Li et al., 2021; Zhang et al., 2021; Tavirani et al., 2019). Oxytocin (OXT) was reported to be upregulated in NAFLD via bioinformatics (Park et al., 2007), which was consistent with our result. Thus, we reported for the first time that FABP4, CPOX, TLR2, and OXT can be utilized as potential diagnostic biomarkers in NAFLD, which may serve as the basis for further research.

CPOX, which is the enzyme that catalyzes the sixth step in the heme biosynthesis pathway, has been found among the four major regulators of FAM. While its primary function relates to the metabolism of porphyrins, CPOX is associated with oxidative stress in the liver and abnormal lipid metabolism. Heme is an important prosthetic group necessary for cytochromes and other hemoproteins, which participate in mitochondrial respiration and transfer electrons. Heme dysregulation leads to disruption of mitochondrial processes, ROS generation, and decreased activity of antioxidants. This, in turn, increases the ‘second hit’ in NAFLD development. Heme dysregulation is also related to the occurrence of inflammation and macrophage activation in the liver. Concerning our study, the elevated level of CPOX in NAFLD patients could be explained by the compensatory increase of this protein due to mitochondrial or oxidative stress caused by the excess of lipids in NAFLD development.

Kupffer cells, which are macrophages located in the liver, play a crucial role in the liver’s innate immune system. Research (Huang et al., 2010) has shown that the degree of macrophage buildup in liver tissue is directly related to the severity of NAFLD. It was found that complete clearance of macrophages or Kupffer cells in multiple NASH models prevented the occurrence of inflammation, steatosis, and fibrosis (Lanthier et al., 2011; Tosello-Trampont et al., 2012; Cusi, 2012). However, the mechanisms governing the contribution of macrophages to NAFLD progression are complicated and incompletely elucidated. Macrophages may be activated by free fatty acids derived from adipose tissue by means of TLR signaling pathways. Specifically, palmitate and TLR2 may synergically induce the inflammasome activation of macrophages, while palmitate interacts with TLR4/MD2 complex to stimulate ROS production in inflammatory macrophages (Kim et al., 2017; Miura et al., 2013; Shi et al., 2006; Böhm et al., 2013). Furthermore, trans-fatty acids and lipid peroxides from an unbalanced diet may also prompt the activation of macrophages (Stienstra et al., 2010). In addition to their role in inflammation, macrophages impact fatty acid metabolism. They inhibit the activity of peroxisome proliferator activator receptor α through IL-1β-dependent inhibition and decrease fatty acid oxidation in hepatocytes by accelerating the accumulation of triglyceride in hepatocytes, thereby forming a positive-feedback loop between macrophage activation and fatty acid metabolism disorder to enhance NAFLD development (Stienstra et al., 2010). In addition, TNF was reported to be involved in mediating macrophage-dependent weakened fatty acid oxidation and increased triglyceride accumulation. This observation is supported by findings that TNF blockade was observed to ameliorate the two processes in the rat macrophage-hepatocyte co-cultured model (Lanthier et al., 2011). It was found that patients with NAFLD in FAM subtype B exhibit higher infiltration levels of macrophages, indicating that these patients are likely to have more severe hepatic steatosis and may be potentially treatable with TNF blockade. Our subsequent assessment of the pivotal FAM regulatory pattern also verified that both patients with NAFLD in FAM subtype B and gene subtype B have higher FAM scores. This suggests a more active FAM regulatory pattern or stronger FAM regulatory activity in these two subtypes. Furthermore, higher expression levels of lipase A (LIPA) in both FAM subtype B and gene subtype B correspondingly revealed that the hydrolysis of cholesteryl esters and triglycerides is more active. This heightened activity may result from compensatory processes aimed at addressing the over-deposition of triglycerides in both FAM subtype B and gene subtype B.

Some limitations in this study need to be mentioned. First of all, the bioinformatics analysis was mainly done using one GEO database (GSE89632) with a small number of samples (24 control samples and 39 NAFLD samples). Even though the findings were further validated in three additional datasets (GSE48452, GSE66676, and GSE135251), as well as verified experimentally in HepG2 cells and in a mouse model of high-fat diet, more extensive multicentric cohort studies are required to confirm the effectiveness of the four-gene signature (FABP4, CPOX, TLR2, and OXT). Second, the definition of the FAM subtypes and gene subtypes relied solely on transcriptomic studies. The use of multi-omics approaches (such as proteomics, metabolomics, or epigenomics) may provide additional information regarding the molecular diversity of NAFLD. Third, while we observed that subtype B had higher macrophage infiltration and FAM score, the clinical and biological importance of this observation (for example, if it is a candidate for anti-TNF therapy) remains to be elucidated. Fourth, the current *in vitro* (palmitic acid-stimulated HepG2 cells) and *in vivo* (high-fat diet mouse model) studies were relatively basic. More advanced experiments may be used for further mechanistic investigations, such as gene knockdown, overexpression, CRISPR/Cas9 editing, and conditional knockout animals, which are necessary to clarify the exact roles of CPOX, FABP4, TLR2, and OXT in fatty acid metabolism and NAFLD pathogenesis. Finally, while machine learning algorithms (Random Forest) and consensus clustering were used in this study, the developed nomogram has yet to be validated in an independent cohort.

Future studies should validate the diagnostic utility of the identified biomarkers in prospective cohorts and explore their functional roles through targeted gene manipulation. Multi-omics approaches and subtype-specific therapeutic strategies are warranted to advance personalized management of NAFLD.

## 5 Conclusion

In this present study, we managed to develop a diagnostic model to predict the occurrence of NAFLD utilizing four pivotal FAM regulators (FABP4, CPOX, TLR2, and OXT). Two distinct FAM subtypes were also identified in NAFLD based on pivotal FAM regulators. FAM subtype B was characterized by higher macrophage infiltration and more severe FAM disorder, suggesting a potential vulnerability to anti-inflammatory strategies such as TNF blockade, which warrants future investigation. Collectively, these findings provide a framework for subtype-guided diagnostic and therapeutic strategies in NAFLD.

## Data Availability

The datasets presented in this study can be found in online repositories. The names of the repository/repositories and accession number(s) can be found in the article/Supplementary Material.
