# Supplementary material for: Significance of fatty acid metabolism regulators in the diagnosis and subtype classification in non-alcoholic fatty liver disease
Source: Front Cell Dev Biol. 2026 Jun 19;14:1871166. doi: 10.3389/fcell.2026.1871166 (PMC13328353; doi:10.3389/fcell.2026.1871166)
Supplement: Supplementary file 2 [file DataSheet3.docx]

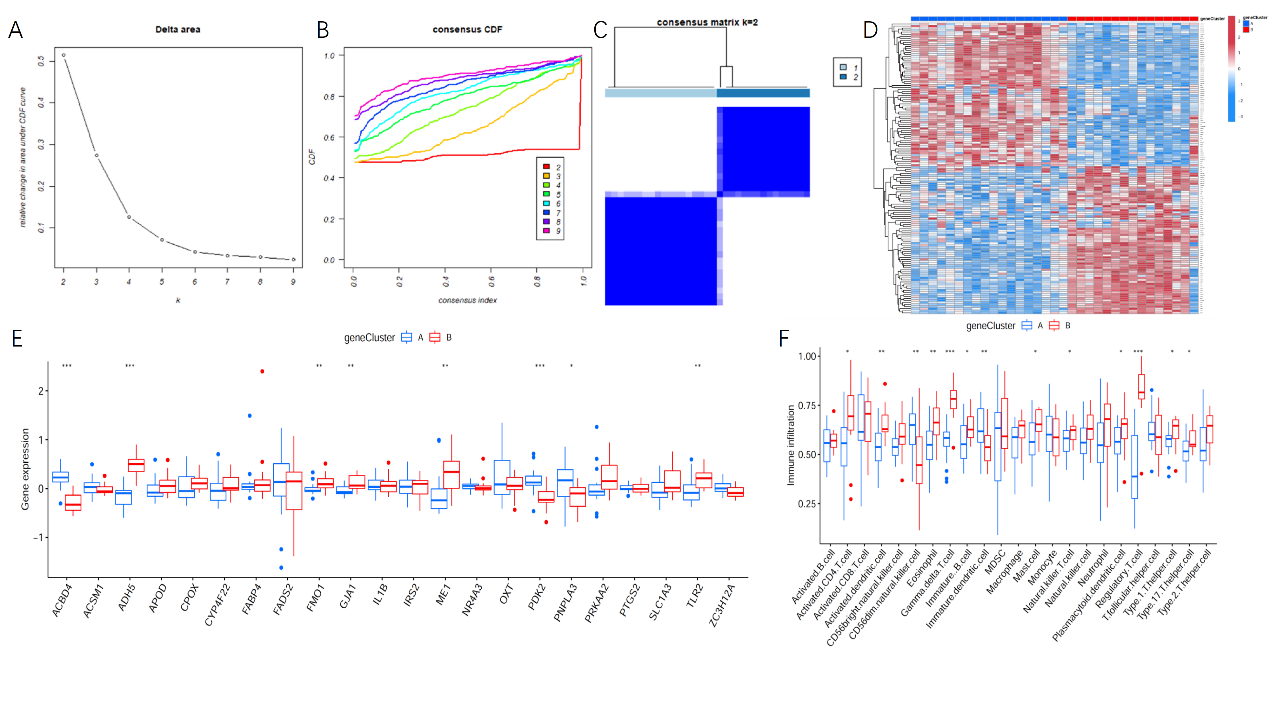


Fig S1. Consensus clustering of the 142 PAM-related DEGs in NAFLD. (A) Relationship between the relative changes in the area under the CDF curve and the numbers of the molecular subgroups (K value). (B) Consensus CDF of the different numbers of clusters. The X-axis represents the consensus index. (C) Heat map of the consensus matrix when the NAFLD samples are clustered into two molecular subgroups (K=2). (D) Expression heat map of the 142 PAM-related DEGs in gene clusterA and gene clusterB. (E) Differential expression histogram of the 22 significant PAM regulators in gene clusterA and gene clusterB. (F) Differential immune cell infiltration between gene clusterA and gene clusterB. *p < 0.05, **p < 0.01, and ***p < 0.001.
